# Determination of an Optimal Standardized Uptake Value of Fluorodeoxyglucose for Positron Emission Tomography Imaging to Assess Pathological Volumes of Cervical Cancer: A Prospective Study

**DOI:** 10.1371/journal.pone.0075159

**Published:** 2013-11-12

**Authors:** Ying Zhang, Jing Hu, Hong-Jun Lu, Jian-Ping Li, Ning Wang, Wei-Wei Li, Yong-Chun Zhou, Jun-Yue Liu, Sheng-Jun Wang, Jing Wang, Xia Li, Wan-Ling Ma, Li-Chun Wei, Mei Shi

**Affiliations:** 1 Department of Radiation Oncology, Xijing Hospital, The Fourth Military Medical University, Xi'an, China; 2 Department of Nuclear Medicine, Xijing Hospital, The Fourth Military Medical University, Xi'an, China; 3 Department of Pathology, Xijing Hospital, The Fourth Military Medical University, Xi'an, China; 4 Department of Radiology, Xijing Hospital, The Fourth Military Medical University, Xi'an, China; Rajiv Gandhi Centre for Biotechnology, India

## Abstract

**Purpose:**

To determine the optimal standardized uptake value (SUV) of ^18^F-fluorodeoxyglucose (^18^F-FDG) for positron emission tomography (PET) imaging, at which the PET-defined gross tumor volume (GTV_PET_) best matches with the pathological volume (GTV_PATH_) in the cervical cancer.

**Materials and Methods:**

Ten patients with the cervical cancer who underwent surgery were enrolled in this study. The excised specimens were processed for whole-mount serial sections and H-E staining. The tumor borders were outlined in sections under a microscope, histopathological images were scanned and the GTV_PATH_ calculated. The GTV_PET_ was delineated automatically by using various percentages relative to the maximal SUV and absolute SUV. The optimal threshold SUV was further obtained as the value at which the GTV_PET_ best matched with the GTV_PATH_.

**Results:**

An average of 85±10% shrinkage of tissue was observed after the formalin fixation. The GTV_PATH_ was 13.38±2.80 cm^3^ on average. The optimal threshold on percentile SUV and absolute SUV were 40.50%±3.16% and 7.45±1.10, respectively. The correlation analysis showed that the optimal percentile SUV threshold was inversely correlated with GTV_PATH_ (p<0.05) and tumor diameter (p<0.05). The absolute SUV was also positively correlated with SUV_max_ (p<0.05).

**Conclusion:**

The pathological volume could provide the more accurate tumor volume. The optimal SUV of FDG for PET imaging by use of GTV_PATH_ as standard for cervical cancer target volume delineation was thus determined in this study, and more cases are being evaluated to substantiate this conclusion.

## Introduction

The fluorine-18 fluorodeoxyglucose positron emission tomography (^18^F-FDG-PET) is a noninvasive three-dimensional imaging procedure that uses a glucose analogue as metabolic tracer. The FDG uptake increases in the cells with high metabolic rate, especially in tumor region, which appears as high-uptake hot spots in FDG-PET image. This imaging technique has been extensively evaluated and widely used for early diagnosis, staging, confirmation of lymph node metastasis and radiotherapy outcome in the cervical cancer [1], [2], [3]. Among these, the accuracy of PET/CT imaging in the diagnosis of gynecologic cancers including cervical cancer is higher than other conventional imaging methods [2].

It is well known that radiotherapy is a treatment of choice for cervical cancer, which can even be cured by radiotherapy alone. Delineation of tumor target volumes accurately has an important significance to achieve precise radiotherapy in the three-dimensional era of radiotherapy. Though CT and MRI have been widely used in radiotherapy planning, it is essential to acquire more information of tumor volume. Presently, it is difficult to discriminate normal soft tissue and tumor border on CT images, even in the contrast-enhanced CT. In this regard, MRI is better than CT for primary tumors and adjacent soft tissue involvement in the pelvis. But, ^18^F-FDG-PET can provide more metabolic activity information of tumor tissue in addition to anatomical tumor characterization. It demonstrated that ^18^F-FDG-PET could measure ‘metabolic volume’ to select the best candidates for radiotherapy [2], [3]. It is clear that ^18^F-FDG-PET may be an advanced imaging technique for target localization and radiation dose calculation for intracavitary brachytherapy and sophisticated external radiation treatment [4], [5]. Furthermore, interobserver variability was effectively decreased by using PET/CT scan in treatment planning and evaluation of response for the cervical cancer [6].

However, it is an unsolved and critical question on how to obtain PET images that will provide tumor target volume accurately, as PET/CT is being currently used more frequently in clinical cancer radiotherapy. Compared with the gross tumor volume (GTV) on CT, the volume of GTV_PET_ changed in two of eight cases of the gynecological cancers. The radiotherapy planning had to be revised because the volume of GTV_PET_ varied about 20±10% [7]. The tumor volumes based on PET/CT were also affected by the threshold level of standardized uptake value (SUV). The different threshold of SUV caused significant differences in radiation dose to target and normal tissues [8]. Identification of an optimal PET segmentation method could reduce these errors and interobserver variability in GTV delineation, and potentially allow for reduction in GTV expansions. Data from studies indicated no consensus in optimal SUV selection when PET/CT was utilized to outline radiotherapy target volume, especially in lung cancer, head and neck tumor [9], [10], [11].

In this study, we have compared the volume based on the PET/CT functional imaging information of the cervical cancer with the pathologic volume reconstructed by a technique of whole-mount serial sections. The aim of this study was to explore feasibility of using the reconstructed pathologic volume as the standard to determine the optimal SUV cutoff-value of PET/CT images and to provide an experimental basis for delineating biological target volume of cervical cancer. At the same time, the range of microscopic extension in the different directions of the cervical cancer was observed under a microscope to observe boundary of clinical target volume. To our knowledge, this is the first report on correlation of image-based radiotherapy target volume and reconstructed pathologic volume of the cervical cancer.

## Materials and Methods

### Patients group

Ten patients with histologically confirmed cervical cancer were selected for the present study at the Xijing Hospital between March 2010 and November 2010. The eligibility criteria were included as invasive squamous cell carcinoma with FIGO clinical stageIB-IIB. The patients were free of diabetes or other diseases that could affect the results of FDG-PET/CT, and had not undergone any cancer-related treatments previously. All patients underwent radical hysterectomy and pelvic lymphadenectomy and the ^18^F-FDG-PET/CT scans on the day before surgery. The protocol was approved by Hospital's Protection of Human Subjects Committee (Xijing Hospital, Fourth Military Medical University, Clinical Ethics Committee), and written consent was taken from all the patients.

### PET/CT scan and gross target volume in PET image (GTV_PET_) definition

The FDG-PET/CT scan was performed with a hybrid PET/CT scanner (Biograph 40; Siemens Medical Solutions, Malvern, PA). All patients were asked to fast for at least 6 h before the PET examination and their blood glucose levels were checked before injecting the tracer. About forty to sixty minutes after intravenous injection of ^18^F-FDG (5.55–7.40MBq/kg), the CT image scan was extended from L3 to tuber ischiadicum by using 64-slice helical CT acquisition (140 KV, 90 mA, a slice thickness of 3 mm, a 50 cm displayed field of view, and a rotational speed of 0.75 s per rotation) and emission images were acquired covering the same axial range. The PET images were reconstructed with CT-derived attenuation correction using ordered-subset expectation maximization software.

**Figure 1:**
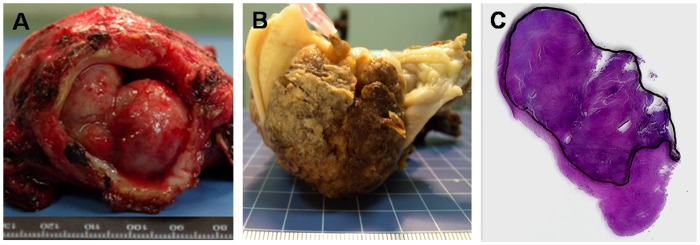
Determination of the optimal standardized uptake value (SUV) cut-off value. Data from case 6 is shown as a representative example. The serial gross tumor volumes on positron emission tomography (GTV_PET_) measured on the basis of each cut-off value (A, SUV = 1.5, 2.5, 3.5, 4.5 and 5.5; B, % SUV = 10, 20, 30, 40 and 50) are plotted according to the threshold relative to the absolute SUV (C) or percentages of maximal SUV (D). The optimal cut-off value is determined by the linear approximation with the best agreement between the GTVPET and pathologically determined GTV (GTV_path_).

The PET/CT images were reviewed by two experienced nuclear medicine physicians on Xeleris workstation. The tumor was characterized by an increased FDG uptake beyond expected normal tissue uptake (standardized uptake value (SUV) being >2.5). The gross target volume confirmed by absolute SUV level (GTV_PET(SUV)_) was calculated automatically by the True-D Software at various cut-off level of the absolute SUV, from 2.5 to 12.5 at 0.5 intervals (representative PET images at absolute SUV value of 2.5, 3.5, 4.5, 5.5, 6.5 and 7.5 shown in Fig. 1A). The gross target volume confirmed by percentile level of SUV (GTV_PET(%SUV)_) was calculated by same way at different percentile thresholds of the maximal SUV, from 10% to 60% at 5% interval (representative PET images at 10%, 20%, 30%, 40%, 50% or 60% percentile thresholds of the maximal SUV are shown in Fig. 1B).

### Pathology procedure

**Figure 2:**
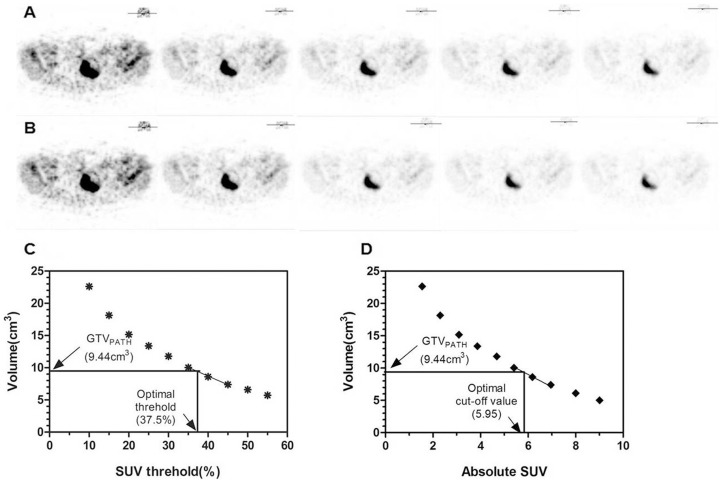
A representative sample showing measurement of pathologic gross tumor volume of cervical cancer. The surgical specimen is oriented to the *in vivo* geometry and bisected in the transverse plane. The dimensions of the sample are measured before (A) and after formalin fixation (B). The fixed specimen is then sectioned into 4-6μm slices. In the sections stained with hematoxylin and eosin, the tumor-containing region is delineated by black line (C).

All the patients underwent radical hysterectomy and pelvic lymphadenectomy. The tumor maximum diameter and vertical diameter were measured and recorded (Table 1). Once the specimens were obtained, tumor and cervix were measured and fixed using 10% formalin solution for 24h at room temperature. The maximum diameter and vertical diameter of fixed tumor were recorded again to calculate volume reduction (Fig. 2A, B.). Next, the specimens were cut into serial slices of 4 mm thickness. The slices were again cut into 4μm thick histological sections with a microtome (Microm HM 450; GMI, Ramsey, MN) after routine dehydration and paraffin-embedding procedure. The 4μm thick sections were processed for hematoxylin and eosin (HE) staining. Under a light microscope, the tumor border on the HE-stained histological slides was outlined by an experienced pathologist blinded to the imaging results (Fig. 2C). The regions of interest of the HE stained sections were captured by a digital camera with a ruler. The captured images were then dealt with Adobe Photoshop software and outlined tumor areas were calculated by histogram function of software layers of the pixel sections. Finally, the pathologic gross target volume (GTV_PATH_) was calculated according to summation formula, i.e. GTV_PATH_  =  Σ area × thickness/R, in which R was defined as the ratio of the sample dimensions before and after formalin fixation.

**Table 1:** Summary in tumor size of pre- and post-fixation, retraction ratio of corresponding area and volume, and actual pathologic volume of ten cervical cancers.

Case No.	D_long_(cm)	D_short_(cm)	d_long_ (cm)	d_short_(cm)	Area retraction (cm^2^)	Volume retraction (cm^2^)	GTV_path_(cm^3^)	Actual GTV_path_(cm^3^)
1	1.2	0.8	1.1	0.8	0.92	88	1.04	1.18
2	1.5	1.1	1.45	1.1	0.97	95	1.76	1.85
3	2.8	1.3	2.55	1.2	0.85	78	3.64	4.67
4	2.2	1.8	2.15	1.8	0.98	97	4.34	4.47
5	3.8	1.4	3.1	1.3	0.75	65	4.89	7.52
6	4.1	1.8	3.7	1.6	0.80	72	6.80	9.44
7	3.4	2.0	3.1	1.9	0.90	86	12.13	14.10
8	4.9	2.4	4.7	2.3	0.91	87	13.42	15.43
9	5.2	1.6	5.0	1.4	0.93	90	20.84	23.15
10	5.0	2.4	4.9	2.3	0.95	92	24.04	26.13

*Abbreviations:* D_long_ =  long diameter on transverse plane before formalin fixation; D_short_ =  short diameter on transverse plane before formalin fixation; d_long_ =  long diameter on transverse plane after formalin fixation; d_short_  =  short diameter on transverse plane after formalin fixation; Area retraction  =  tumor transverse plane reduction after formalin fixation, which is calculated according to the formula (d_long_× d_short_)/(D_long_×D_short_); Volume retraction  =  tumor volumetric retraction after fixation, which equals (area retraction); GTV_path_  =  pathologic tumor volume.

### Determination about optimal cut-off value of SUV

The GTV_PATH_ with the GTV_PET(SUV)_ and GTV_PET(%SUV)_ were compared case by case. A coordinate system was established with the vertical axis of absolute SUV or percentile SUV threshold and abscissa of GTV_PET_. A linear approximation could be seen between the adjacent two points in coordinate system. The GTV_PET(SUV)_ and GTV_PET(%SUV)_ were obtained best in agreement with GTV_PATH_ by a first-order linear approximation (Fig. 1). The optimal cut-off value of SUV was the absolute or percentile values of SUV when the GTV _PATH_ was equal to the GTV_PET_.

### Statistical methods

All data were statistically analyzed by SPSS software (version 13.0) in this study. Wilconxon signed-rank test was used to compare GTV_PET(SUV)_ and GTV_PET(%SUV)_ with the corresponding GTV_PATH._ Spearman correlation test was used to analyze relationship between optimal cut-off absolute SUV or percentile SUV threshold, tumor maximum diameter and GTV_PATH_. The cases satisfying p<0.05 were considered as statistically significant for all comparison tests in this study.

## Results

### Patient details

All the patients were confirmed with the invasive squamous carcinoma of cervix. The median age of 10 patients was 40 years (ranging 32–55 years). The clinical stage (FIGO): 2 of I_B_, 3 of II_A_ and 5 of II_B_. All patients exhibited abnormal radioactive uptake in tumor (SUV>2.5). No patient showed abnormal lymph nodes in the pelvic cavity.

### Retraction of surgical specimen after fixation

The volume of tissue changed during the pathological procedure. A notable shrinkage of tissue occurred during the formalin fixation procedure. The mean volume of tumor after fixation was about 85%±10% (ranging 65–97%) of the original tumor volume (Table 1).

### Optimal cut-off value of SUV

The mean SUV_max_ and SUV_mean_ were 18.38±8.85 and 7.88±2.85, respectively. The SUV values varied among patients. The range of absolute SUV threshold was from 3.8 to 13.0 and the mean of optimal absolute SUV was 7.45±1.10. The percentile SUV threshold was from 24.9% to 55% and the mean of optimal percentile SUV was 40.50%±3.16% (Table 2).

**Table 2:** Summary in SUVs, GTVs and Diameters of ten cervical cancers.

Case No.	SUV_max_	SUV_mean_	Optimal cut-off SUV	Optimal SUV threshold(%)	GTVpath (cm^3^)	Diameter (cm)
1	10.72	5.29	4.79	53.2	1.18	1.2
2	20.33	8.73	13.0	55	1.85	1.5
3	27.77	10.16	11.6	42.2	4.67	2.8
4	23.35	10.64	5.4	49.0	4.47	2.2
5	9.03	4.46	3.8	35.5	7.52	3.8
6	15.44	10.07	5.95	37.5	9.44	4.1
7	9.03	4.46	6.2	34.1	14.10	3.4
8	27.55	9.58	11.8	44.1	15.43	4.9
9	31.59	11.01	8.1	24.9	23.15	5.2
10	9.0	4.42	3.8	29.5	26.13	5.0
mean±SD	18.38±8.85	7.88±2.85	7.45±1.10	40.50±3.16	13.38±2.80	3.41±0.46

*Abbreviations:* SUV_max_  =  maximum standardized uptake value; SUV_mean_  =  mean standardized uptake value; GTV_path_  =  pathologic gross tumor volume.

**Figure 3:**
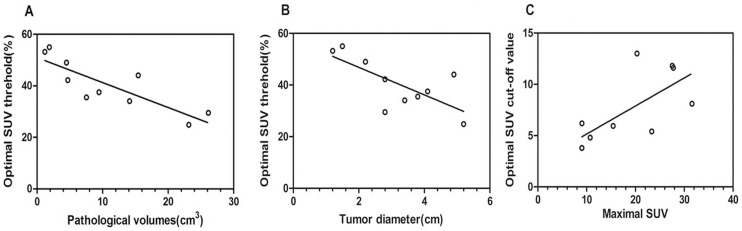
Correlation analysis of presented data. A correlation is shown between optimal percentile SUV threshold and the gross tumor volumes determined by pathology (A), or tumor diameter (B), and correlation shown between the maximal SUV and the optimal absolute SUV (C).

The correlation of SUV values with tumor pathologic volume and diameter was further analyzed. The optimal percentile SUV threshold was inversely correlated with GTV_PATH_ (r = −0.8424, *p* = 0.0037) (Fig. 3A) and tumor diameter (r = −0.8085, *p* = 0.0072) (Fig. 3B).

There was a significant difference of the percentile SUV among the different tumor diameters or different pathologic volumes. When they were divided tumor into three groups by diameter, i.e. <2.5 cm, 2.5–4.5 cm, and >4.5 cm, the optimal percentile SUV was 52.4%±3.08%, 37.33%±3.54% and 32.83%±10.02%, respectively (*p* = 0.0126). The percentile SUV threshold for tumors of different pathologic volumes was 32.83%±10.02% for tumors being >15 cm^3^, 35.70%±1.71% for tumor volume ranging between 5–15 cm^3^, and 49.9%±5.67% for tumors being <5 cm^3^, respectively (*p* = 0.0224).

On the other hand, there was no significant correlation between the optimal absolute SUV value and tumor diameter (r = −0.07295, *p* = 0.8382) or volume (r = −0.07295, *p* = 0.8382). The optimal absolute SUV was 7.73±4.57 for tumor diameter being <2.5 cm, 6.89±3.32 for diameter being between 2.5 cm and 4.5 cm, and 7.90±4.00 for diameter being >4.5 cm, respectively (*p* = 0.9340). It was 7.90±4.00 for tumors of size >15 cm^3^, 5.32±1.32 for tumors of size 5–15 cm^3^, and 8.70±4.21 for tumors of size <5 cm^3^ (p  = 0.4829). The absolute SUV was positively correlated with SUV_max_ (r = 0.6921, *p* = 0.0306) (Fig. 3C). However, no significant correlation was identified between the optimal percentile SUV and SUV_max_ (r = −0.07295, *p* = 0.8382).

## Discussion

The novelty of the present piece of study is that pathological volume could provide the more accurate tumor volume and the PET-defined gross tumor volume (GTV_PET_) matched best to the pathological volume(GTV_PATH_) in the cervical cancer. By using whole-mount serial section technique, it revealed that shrinkage rate of tumor volume was from 65% to 97% (mean 85±10%) after pathological process and actual volume of tumor was obtained by reconstruction procedure. Data of this study indicate that the actual tumor volume could be considered as more accurate volume and be potentially used as “the gold standard” to compare PET volume and further to determine the optimal SUV value in PET images for radiotherapy of cervical cancer.

The common methods confirming SUV value on PET imaging are roughly divided into three types. They included one studying the phantom, one comparing with other types of image, and one comparing with tumor size determined by pathological volume. Through phantom studies, the researchers found an optimal SUV thresholds for PET as between 36% to 44% for sphere volume larger than 4 mm^3^ and the exact value might depend on the source-to-background (S/B) ratios [12]. The optimal SUV threshold changed while it was compared with the static phantom and moving phantom [11], [13], [14]. When FDG-filled different diameter phantom was utilized, the appropriate threshold level depended on lesion size [8] and 40–50% of the maximum SUV seemed to be appropriate for GTV contouring of sphere tumors with homogenously distributed ^18^F-FDG [15]. Besides, the FDG uptake in a tumor tissue showed a more gradual decrease toward the tumor edge and might be also affected by other blurring effects, such as respiratory motion, given the FDG-avid volumes in phantom studies were demarcated sharply.

To obtain the best SUV value, there were many studies comparing PET image with other type of image, especially CT, which was most commonly used to delineate the target volume for radiotherapy planning. Nestle U *et al* compared the GTVs with confirmation of four methods in 25 patients with primary non small cell lung carcinoma (NSCLC), and found that a threshold of 40% of the maximum standardized uptake value was not suitable for target volume delineation [16]. Biehl KJ *et al* reported that a single standardized uptake value threshold was not adequate for NSCLC [17]. In a later observation, Hong R *et al* recommended a method using SUV being ≥2.5 for radiotherapy planning in NSCLC by comparing with CT [18]. Researchers contoured the GTVs with six different “observers”, i.e. two radiation oncologists, two nuclear medicine physicians and two radiologists, by using SUV cutoffs of 2.5 and 3.5 and 40% SUV_max_ in 6 NSCLC patients. They found an SUV contour of 2.5 was most closely correlated with the mean GTV defined by the interobservers [19]. In other types of tumors such as esophageal cancer, a threshold of approximate 2.5 yielded the highest conformality index and approximate CT-based GTV at the epicenter [20]. Obviously, researchers might get various SUV suitable for different sizes and types of tumor. Currently, CT or MRI was widely used in contoured tumor and the image was easier obtained. However, both CT and MRI also exhibited disadvantages in delineating tumor border accurately, which mostly depended on personal experience in a large extent and tumor volume on CT was often overestimated.

Furthermore, some researchers calculated the actual tumor volume and used it as a reference standard to find best SUV value. Burri RJ *et al* studied the correlation of PET SUV with pathologic specimen size in patients with head-and-neck cancers. They drew a conclusion that the SUV40 method might appear to offer balance between accuracy and reducing risk of underestimating tumor extent [21]. In the esophageal cancer, the mean optimal threshold was 23.81%±11.29%, but the optimal threshold was different with various tumor sizes. An SUV cutoff of 2.5 provided the closest estimation when the length of PET matched the length of pathology [22]. In NSCLC researches, Yu *et al* found an optimal threshold of 31%±11% and an absolute SUV of 3.0±1.6, respectively [9]. Similarly, the threshold SUV varied with different tumor diameter (>5 cm, 3–5 cm, and <3 cm), but the difference was not significant statistically. In those studies, the pathologic specimen was used as the “gold standard”, which was recommended to reflect actual tumor volume more accurately.

In previous feasibility studies, a threshold of 40%–50% of maximum SUV was found to be a reliable correlate to tumor volume measured by CT [7], when the use of PET/CT was evaluated in radiation therapy planning. However, SUV on PET might vary with different anatomical sites, organizational characteristics and tumor activity in different type of cancer. In a retrospective study, Nguyen et al compared the SUV_max_ of FDG PET in four different sites and found that no common SUV_max_ threshold exists [23]. In another retrospective study, Chung et al measured the metabolic tumor volume(MTV) by integrated FDG-PET/CT imaging and used a fixed threshold of SUV 2.5 and found the MTV was good correlated with tumor volume calculated from surgical specimen [24]. This result was similar with previous report in the lung cancer [25]. However, the results of our prospective study further indicated an increased optimal threshold SUV or absolute SUV in cervical cancer compared with that of esophageal cancer or NSCLC, and the difference might mainly result from the less tumor motion in cervical cancer.

In this study, data showed that optimal percentile threshold SUV varied with changes of tumor volume. The optimal percentile threshold SUV reduced when the tumor volume increased, although the differences were not significant statistically. In contrast, no correlation was observed between the absolute SUV and tumor size of the cervical cancer. The optimal percentile threshold SUV showed an inverse correlation with GTV_PATH_ and tumor diameter in our study, which was well consistent with previous report in NSCLC [9]. Another research indicated that the optimal PET threshold was inversely correlated with the GTV_CT_ [17]. Besides, it also found an increased accuracy by using a threshold function, relative to a constant threshold in phantom [26]. One study recently showed that 40% threshold was suitable for calculating the volume of any lesion with diameter being >1.83 cm, 60% for diameter being >1.35 cm but <1.83 cm, and 75% for diameter being <1.35 cm by phantom observation [27]. The overall results indicate that a fixed SUV cut-off might not be appropriate to all tumor volumes.

To the best of our knowledge, there are few research reports comparing tumor volumes determined by different imaging modalities with surgical specimens in the cervical squamous cell tumors, and we thus concluded that GTVs derived from the PET images matched those from microscopy more accurately [28]. This is a first observation in cervical cancer for PET SUV determination, and the data of this study could provide a basis and reference for SUV selected for PET image in the cervical cancer. In addition, it may need to point out that a limitation of this study was small number of cases, relatively small tumor diameter and tumor volume of samples enrolled. More cases with various tumor volumes and diameters should be included and should be performed for further validation of this study.

## Conclusion

In this study, the SUV of 18F-FDG-PET image was studied by comparing GTV_PET_ and GTV_PATH_ in the cervical cancers. Data analysis indicated that pathological volume provided a more accurate tumor volume. The optimal SUV of PET imaging is thus proposed to use GTV_PATH_ as standard for cervical cancer target volume delineation for improving the radiotherapy planning. Currently, more cervical cancer cases are being investigated to substantiate this conclusion.
